# Enhancing the capability of *Klebsiella pneumoniae* to produce 1, 3‐propanediol by overexpression and regulation through CRISPR‐dCas9

**DOI:** 10.1111/1751-7915.14033

**Published:** 2022-03-17

**Authors:** Xin Wang, Lin Zhang, Shaoxiong Liang, Ying Yin, Pan Wang, Yicao Li, Wee Shong Chin, Jianwei Xu, Jianping Wen

**Affiliations:** ^1^ 12605 Key Laboratory of Systems Bioengineering (Ministry of Education) Tianjin University Tianjin 300072 China; ^2^ 12605 SynBio Research Platform Collaborative Innovation Center of Chemical Science and Engineering (Tianjin) School of Chemical Engineering and Technology Tianjin University Tianjin 300072 China; ^3^ Institute of Materials Research and Engineering Agency for Science, Technology and Research #08‐03, 2 Fusionopolis Way Singapore 138634 Singapore; ^4^ 37580 Department of Chemistry National University of Singapore 3 Science Drive 3 Singapore 117543 Singapore; ^5^ Dalian Petrochemical Research Institute of Sinopec Dalian 116000 China

## Abstract

*Klebsiella pneumoniae* is a common strain of bacterial fermentation to produce 1, 3‐propanediol (1, 3‐PDO). In general, the production of 1, 3‐PDO by wild‐type *K. pneumoniae* is relatively low. Therefore, a new gene manipulation of *K. pneumoniae* was developed to improve the production of 1, 3‐PDO by overexpressing in the reduction pathway and attenuating the by‐products in the oxidation pathway. Firstly, *dhaB* and/or *dhaT* were overexpressed in the reduction pathway. Considering the cost of IPTG, the constitutive promoter P32 was selected to express the key gene. By comparing *K.P*. pET28a‐P32‐*dhaT* with the original strain, the production of 1, 3‐PDO was increased by 19.7%, from 12.97 to 15.53 g l^−1^ (in a 250 ml shaker flask). Secondly, three *lldD* and *budC* regulatory sites were selected in the by‐product pathway, respectively, using the CRISPR‐dCas9 system, and the optimal regulatory sites were selected following the 1, 3‐PDO production. As a result, the 1, 3‐PDO production by *K.P*. L1‐pRH2521 and *K.P*. B3‐pRH2521 reached up to 19.16 and 18.74 g l^−1^, which was increased by 47.7% and 44.5% respectively. Overexpressing *dhaT* and inhibiting expression of *lldD* and *budC* were combined to further enhance the ability of *K. pneumoniae* to produce 1, 3‐PDO. The 1, 3‐PDO production by *K.P*. L1‐B3‐PRH2521‐P32‐*dhaT* reached 57.85 g l^−1^ in a 7.5 l fermentation tank (with Na^+^ neutralizer), which is higher than that of the original strain. This is the first time that the 1, 3‐PDO production was improved in *K. pneumoniae* by overexpressing the key gene and attenuating by‐product synthesis in the CRISPR‐dCas9 system. This study reports an efficient approach to regulate the expression of genes in *K. pneumoniae* to increase the 1, 3‐PDO production, and such a strategy may be useful to modify other strains to produce valuable chemicals.

## Graphical abstract

The 1,3‐PDO production was improved in *K. pneumoniae* by overexpressing the key gene and attenuating by‐product synthesis in the CRISPR‐dCas9 system. This work is efficient and simple, and such a strategy may be useful to be applied to modification of other strains to produce valuable chemicals.

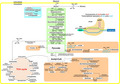

## Introduction

1, 3‐Propanediol (1, 3‐PDO) is an important three‐carbon compound, which is widely used in the chemical, food, cosmetics and other fields (Li *et al*., 2014; Lama *et al*., 2020). 1, 3‐PDO can be synthesized chemically or biologically. Biosynthesis of 1, 3‐PDO has some advantages such as low cost and high efficiency (Lama *et al*., 2017; Lee *et al*., 2018; Chen *et al*., 2020). Under natural conditions, some microorganisms directly use glycerol as a substrate to efficiently synthesize 1, 3‐PDO (Casali *et al*., 2012; Avci *et al*., 2014; Lee *et al*., 2019). The common strains, including *Klebsiella pneumoniae* (Sun *et al*., 2021; Wang *et al*., 2021a,2021b), *Lactobacillus reuteri* (Ju *et al*., 2021a,2021b), and *Clostridium butyricum* (Sedlar *et al*., 2021; Yun *et al*., 2021) have been used to produce 1, 3‐PDO, and this study mainly focused on *K*. *pneumoniae*. In recent decades, researchers have paid much attention to obtain more advantageous strains by genetic engineering (Gonzalez *et al*., 2008; Przystalowska *et al*., 2015; Liu *et al*., 2018; Sun *et al*., 2019), and a more detailed understanding of the metabolic pathway of *K. pneumoniae* is expected to provide a better way to promote the transformation of glycerol into 1, 3‐PDO in this system.

CRISPR‐Cas9 is an immune system of microorganisms and a new gene‐editing tool (Nodvig *et al*., 2018). After mutation of the Cas9 nuclease site (dead Cas9 (dCas9)), CRISPR‐dCas9 did not cut the target gene, but inhibited its expression. Thus, a new gene regulatory tool was developed (Prykhozhij *et al*., 2017; Saifaldeen *et al*., 2021). CRISPR‐dCas9 has advantages over common genome regulatory techniques, such as species restrictions, multiple gene regulatory methods, high gene modification rate and simple simultaneous regulation of multiple genes (Deaner *et al*., 2018; Schwartz *et al*., 2018; Velegzhaninov *et al*., 2020; Minami and Shah, 2021).

Oxidation and reduction reactions are the two metabolic pathways for glycerol as the only carbon source in *K. pneumoniae* (Cheng *et al*., 2004). Glycerol dehydrase (GDHt) and 1, 3‐propanediol oxidoreductase (PDOR) are the key enzymes in the reduction reaction of *K. pneumoniae* to produce 1, 3‐PDO (Cheng *et al*., 2006). The gene *dhaB* encodes GDHt and *dhaT* encodes PDOR. In the reduction pathway, glycerol is first converted to 3‐hydroxypropanal (3‐HPA) and then reduced to 1, 3‐PDO by consuming nicotinamide adenine dinucleotide (NADH) (Chen *et al*., 2005).

In the oxidative pathway, adenosine triphosphate (ATP) and nicotinamide adenine denucleotide (NADH), which are required for cell growth, are produced, along with a series of by‐products, such as acetate, ethanol, lactate, 2, 3‐butanediol (2, 3‐BDO) and so on (Kumar and Park, 2018). This research focuses on the regulation of lactate and 2, 3‐BDO to ultimately improve the production of 1, 3‐PDO under the previous study (Wang *et al*., 1800). *ldhA* and *lldD* encode L‐lactate dehydrogenase (Aguilera *et al*., 2008; Fu *et al*., 2016), while *budA*, *budB* and *budC* are genes in the entire butanediol (BDO) pathway (Kumar *et al*., 2016). Knockout of genes in the lactate pathway and the entire BDO pathways ultimately increases the production of 1, 3‐PDO. However, completely deleting *budO* (whole‐bud operon) may result in heavy carbon metabolic traffic at pyruvate nodes, thereby inhibiting GDHt (Kumar *et al*., 2016). And deletion of *ldhA* could not have a significant improvement in the production of 1, 3‐PDO (Zhou *et al*., 2014). Therefore, in this study, the expression levels of lactate dehydrogenase and 2, 3‐BDO dehydrogenase were reduced by disturbance attenuation, which could not only reduce the consumption of NADH, but also minimize the generation of by‐products, and eventually improve the 1, 3‐PDO production.

**Fig. 1:**
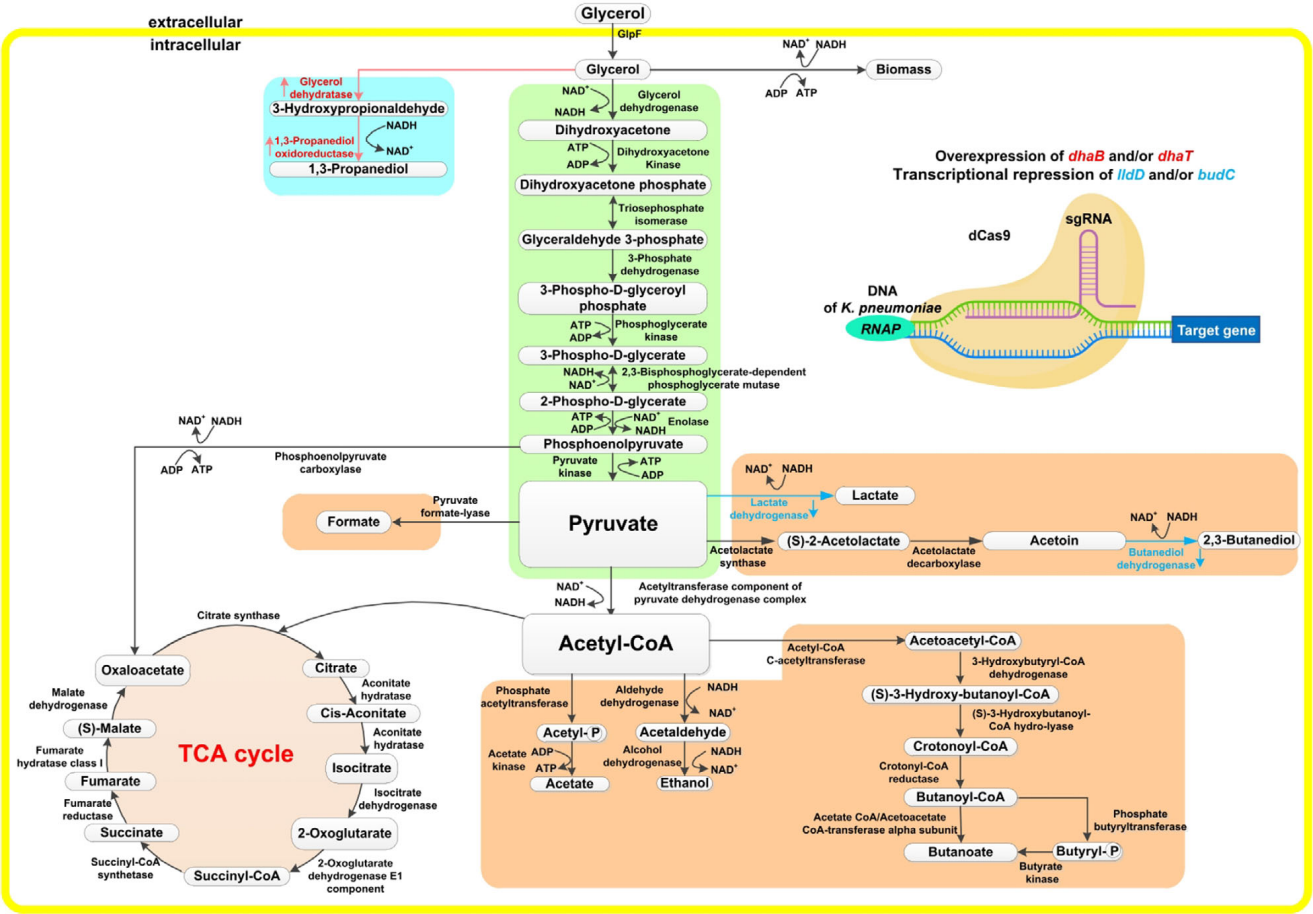
Metabolic pathways of producing 1, 3‐PDO by *K. pneumoniae*. The enzymes marked in red represents the need for overexpression, and the enzymes marked in blue represents the need for regulation.

In this research, the 1, 3‐PDO production by *K. pneumoniae* was investigated by means of overexpression and regulation (as shown in Fig. 1). On the one hand, the plasmids pET28a‐P32‐*dhaB*, pET28a‐P32‐*dhaT* and pET28a‐P32‐*dhaB*‐*dhaT* were constructed, and the influence of overexpressing *dhaB* and/or *dhaT* on the 1, 3‐PDO production was studied. On the other hand, plasmids L1~L3‐pRH2521, B1~B3‐pRH2521 and L1‐B3‐pRH2521 were constructed to explore the effect of *lldD* and *budC* on enhancing the production of 1, 3‐PDO in the oxidation pathway. Finally, the plasmid L1‐B3‐pRH2521‐P32‐*dhaT* was constructed, and the 1, 3‐PDO production by *K.P*. L1‐B3‐pRH2521‐P32‐*dhaT* was increased by 26.1% compared with the original strain. These results will provide ideas on how to improve the 1, 3‐PDO production by *K. pneumoniae* and also give new insights into the genetic modification of other strains.

## Results

### Overexpressing the *dhaB* and/or *dhaT* in the reduction pathway

**Fig. 2:**
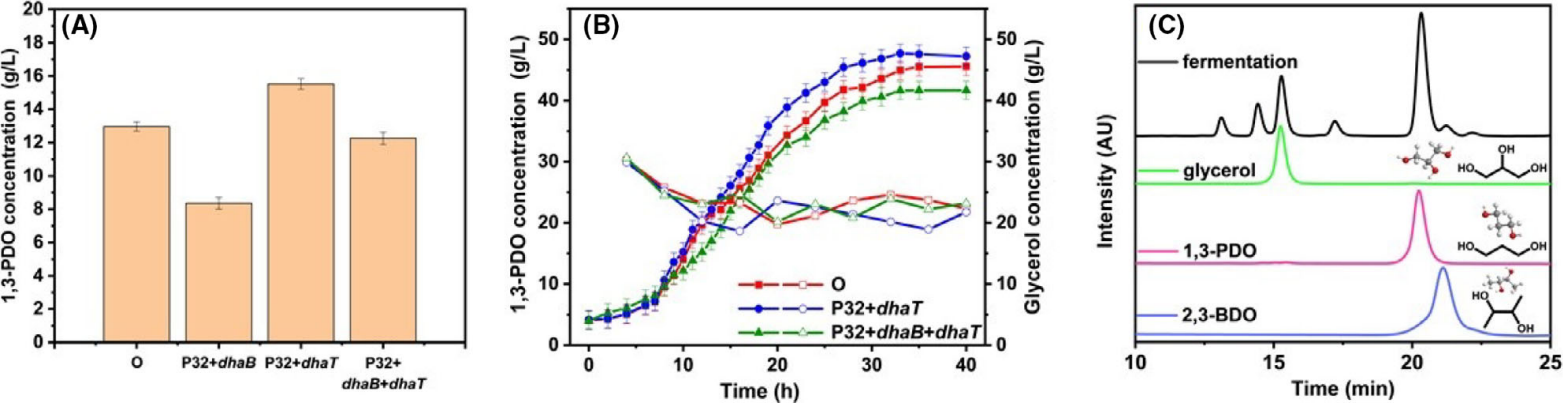
(A) Compare the production of 1, 3‐PDO in the 250 ml shaker for *K. pneumoniae* ATCC15380 (O), *K.P*. pET28a‐P32‐*dhaB* (P32‐*dhaB*), *K.P*. pET28a‐P32‐*dhaT* (P32‐*dhaT*) and *K.P*. pET28a‐P32‐*dhaB*‐*dhaT* (P32‐*dhaB*‐*dhaT*). B. The comparison of the yield of 1, 3‐PDO in the 7.5 L fermentation tank for *K. pneumoniae* ATCC15380, *K.P*. pET28a‐P32‐*dhaT* and *K.P*. pET28a‐P32‐*dhaB*‐*dhaT*. (Solid represents 1, 3‐PDO and hollow represents glycerol.). C. The overlapped chromatograms of glycerol, 1, 3‐PDO and 2, 3‐BDO in zymotic fluid, including the structures. The data represent the mean values of three independent biological replicates, and the error bars represent the standard deviations.

In the process of glycerol metabolism, GDHt is the rate limiting enzyme, which converts glycerol to 3‐HPA, and then is catalysed by PDOR to 1, 3‐PDO (Sankaranarayanan *et al*., 2017). Therefore, some plasmids without the isopropyl‐beta‐D‐thiogalactopyranoside (IPTG) as promoter was constructed to transfer into K. pneumoniae to study the production of 1, 3‐PDO. Figure 2A is a comparison of the 1, 3‐ PDO productions by *K. pneumoniae* ATCC 15380, *K.P*. pET28a‐P32‐*dhaB*, *K.P*. pET28a‐P32‐*dhaT* and *K.P*. pET28a‐P32‐*dhaB*‐*dhaT* in 250 ml shaker. The 1, 3‐PDO production was 12.97, 8.35, 15.52 and 12.25 g l^−1^ respectively. In contrast, only overexpressing *dhaB* was found to reduce the 1,3‐PDO production. In this case, the plasmid pET28a‐P32‐*dhaT* was discovered to be the most effective. In order to further study the effect of *K. pneumoniae* ATCC 15380, *K.P*. pET28a‐P32‐*dhaT* and *K.P*. pET28a‐P32‐*dhaB*‐*dhaT* on the field of 1, 3‐PDO, experiments were conducted in a 7.5 L fermenter (Fig. 2B). The 1, 3‐PDO production by *K.P*. pET28a‐P32‐*dhaT* was found to be the highest at 47.71 g l^−1^, which was only slightly higher than that of the original strain (45.86 g l^−1^). During the entire fermentation process, the concentration of the glycerol as substrate was maintained at 20 g l^−1^. The overlapped chromatograms of glycerol and 1, 3‐PDO during fermentation, including the structures are shown in Fig. 2C. The characteristic peaks at ~ 15.25 and ~ 20.23 min represented the glycerol and 1, 3‐PDO respectively.

### Attenuating the expression of the *lldD* and *budC* in the oxidative pathway

**Fig. 3:**
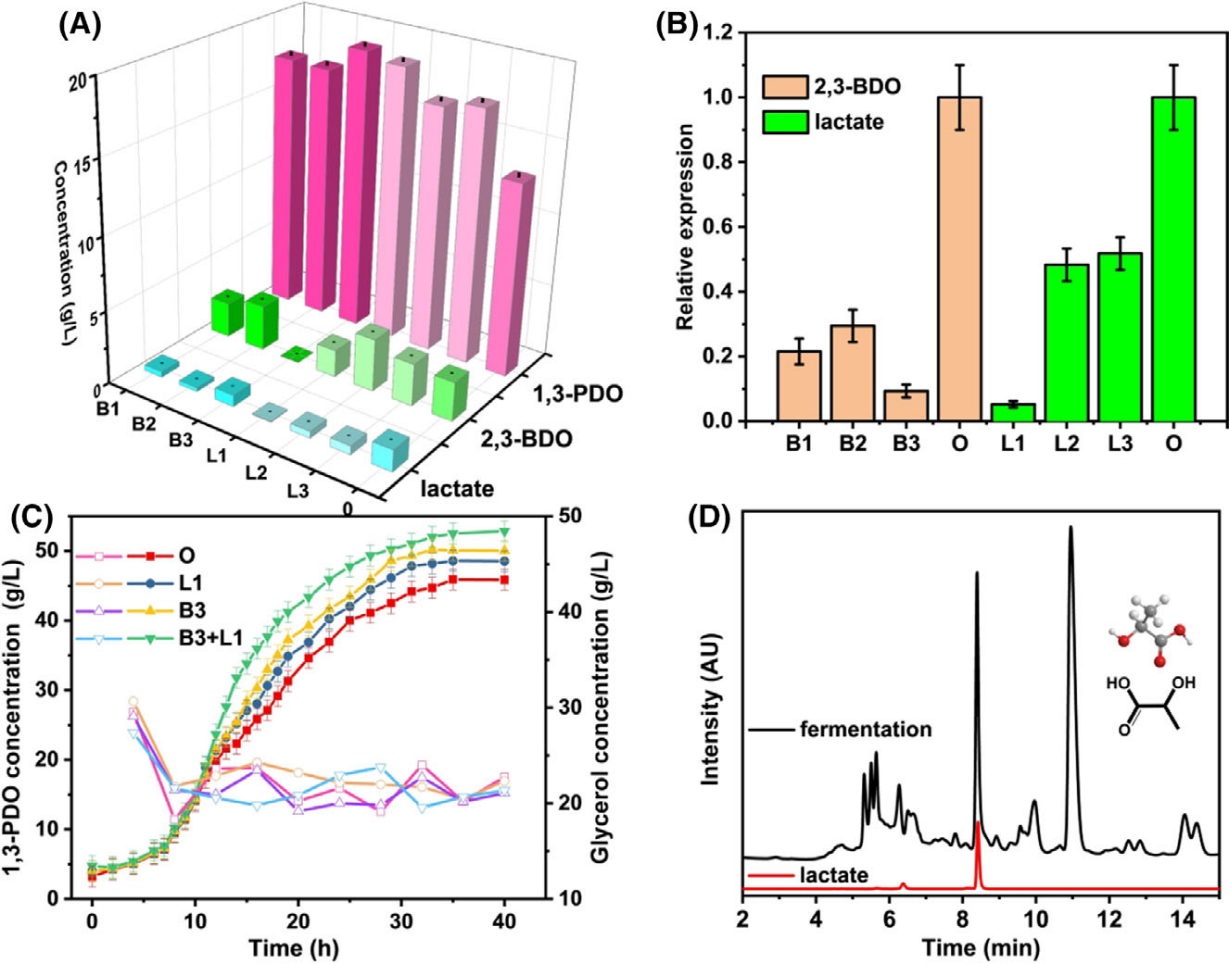
(A) Compare the production of 1, 3‐PDO, 2, 3‐BDO and lactate respectively in the 250 ml shaker for the *K. pneumoniae* ATCC15380 (O), B1 (*K.P*. B1‐pRH2521), B2 (*K.P*. B2‐pRH2521), B3 (*K.P*. B3‐pRH2521), L1 (*K.P*. L1‐pRH2521), L2 (*K.P*. L2‐pRH2521) and L3 (*K.P*. L3‐pRH2521). B. Compare the qRT‐PCR of 2, 3‐BDO and lactate respectively in the 250 ml shaker for the *K. pneumoniae* ATCC15380, B1, B2, B3, L1, L2 and L3. C. The comparison of the yield of 1, 3‐PDO in the 7.5 L fermentation tank for the *K. pneumoniae* ATCC15380, B3, L1 and B3+L1 (*K.P*. B3‐L1‐pRH2521). (Solid represents 1, 3‐PDO and hollow represents glycerol.) D. The overlapped chromatograms of lactate in zymotic fluid, including the structures. The data represent the mean values of three independent biological replicates, and the error bars represent the standard deviations.

According to off‐target effects and specific quality, three sgRNA were designed for *lldD* and *budC* respectively. The best corresponding sgRNA could be analysed by comparing the production pair of lactate and 2, 3‐BDO and the relative expression levels of corresponding genes. Figure 3A shows the 1, 3‐PDO production by *K.P*. L1‐pRH2521 (L1), *K.P*. L2‐pRH2521 (L2), *K.P*. L3‐pRH2521 (L3), *K.P*. B1‐pRH2521 (B1), *K.P*. B2‐pRH2521 (B2), *K.P*. B3‐pRH2521 (B3) and the original strain in 250 ml shaker. It was found that regulating the expression of *budC*, 2, 3‐BDO production was the lowest (0.025 g l^−1^), and 1, 3‐PDO production was the highest (19.16 g l^−1^) for the B3. The lactate of L1 was the lowest (0.052 g l^−1^), and the production of 1, 3‐PDO was the highest (18.74 g l^−1^) when the expression of *lldD* was regulated. The relative expression of B1, B2, B3, L1, L2, L3 and the original strain are shown in Fig. 3B, which are 0.216, 0.295, 0.094, 0.052, 0.483, 0.518 and 1 respectively. It was observed that B1, B2, B3, L1, L2 and L3 revealed the reduced expression of the corresponding genes compared with the original strain. In particular, B3 and L1 had the lowest expression levels, corresponding to the result in Fig. 3A. Compared with completely knockout of lactate dehydrogenase (Zhou *et al*., 2014) and the entire BDO pathways (Kumar *et al*., 2016), the 1, 3‐PDO production by the regulation method has been significantly improved. The combination of L1 and B3 was selected to design L1‐B3‐PRH2521 to obtain higher 1,3‐PDO production.

To further compare the 1, 3‐PDO production after regulating *lldD* and *budC*, a fermentation experiment was carried out in a 7.5 l fermenter. According to Fig. 3C, it can be found that the 1, 3‐PDO production by B3, L1 and L1‐B3 are 50.04, 48.54 and 52.85 g l^−1^ respectively. The 1, 3‐PDO production of L1‐B3 was increased by 15.2% compared with the original strain. Overexpressing *dhaT*, or inhibiting the expression of *budC* and *lldD*, respectively, did not significantly increase the production of 1, 3‐PDO by *K*. *pneumoniae*. Therefore, simultaneous overexpression and inhibition of *K*. *pneumoniae* should be considered to improve the ability of *K*. *pneumoniae* to produce 1, 3‐PDO. Figures 2D and 3C show the overlapped chromatograms of lactate and 2, 3‐BDO in zymotic fluid. The peaks located at ~ 8.43 and ~ 21.11 min could attach to the lactate and 2, 3‐BDO. Through the spectrum, the concentration of lactate and 2, 3‐BDO could be obtained.

### Combining regulation and overexpression

**Fig. 4:**
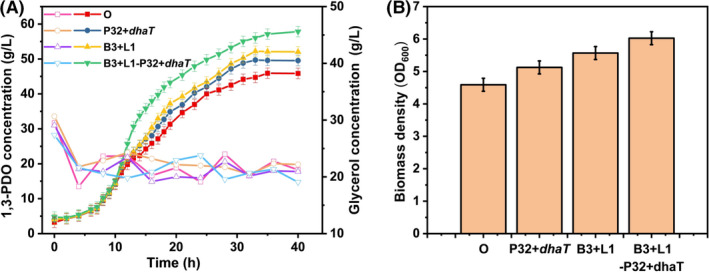
(A) Compare the production of 1, 3‐PDO, 2, 3‐BDO and lactate, respectively in the 250 ml shaker for the *K. pneumoniae* ATCC15380 (O), *K.P*. pET28a‐P32‐*dhaT* (P32‐*dhaT*), B3+L1 (*K.P*. B3‐L1‐pRH2521) and B3+L1‐P32+*dhaT* (*K.P*. B3‐L1‐pRH2521‐P32‐*dhaT*). (Solid represents 1, 3‐PDO and hollow represents glycerol.) B. The cell growth (OD_600_) of O, P32‐*dhaT,* B3+L1 and B3+L1‐P32+*dhaT* in 7.5 L fermenter at 40 h. The data represent the mean values of three independent biological replicates, and the error bars represent the standard deviations.

If gene *dhaT* was overexpressed or *lldD* and *budC* were regulated separately, the increase of 1, 3‐PDO production was limited. In order to further improve the production of 1, 3‐PDO, it is necessary to overexpress *dhaT* in the oxidation pathway and simultaneously attenuate *lldD* and *budC* in the reduction pathway. By comparing the production of 1, 3‐PDO in Fig. 4A, it is found that the 1, 3‐PDO production by *K.P*. L1‐B3‐pRH2521‐P32‐*dhaT* reaches the highest, with the production of 57.85 g l^−1^, which is 26.1% higher than that of the original strain, confirming that the simultaneous overexpression and inhibition of *K*. *pneumoniae* by CRISPR‐dCas9 can improve the ability of producing 1, 3‐PDO. Figure 4B shows the cell growth (OD_600_) of the original strain, *K.P*. pET28a‐P32‐*dhaT, K.P*. B3‐L1‐pRH2521 and *K.P*. B3‐L1‐pRH2521‐P32‐*dhaT* in 7.5 L fermenter at 40 h, which is positively correlated with the production of 1, 3‐PDO. The higher the 1, 3‐PDO production, the greater the OD_600_ of the strain. Among them, the 1, 3‐PDO production by *K.P*. B3‐L1‐pRH2521‐P32‐*dhaT* reached the highest OD_600_ (6.02), which was a 31.15% increase compared with the OD_600_ value of the original strain (4.59).

## Discussion

As reported (Zhang and Xiu, 2009), glycerol disproportionation involves two parallel pathways: reduction pathway and oxidation pathway in the metabolic process of producing 1, 3‐PDO by *K. pneumoniae* (Fig. 1 and Table 1). The reaction of each pathway can be seen from Table 1. The reduction pathway consists of two enzymes: the first enzyme DhaB removes a water molecule from glycerol to produce 3‐HPA; The second enzyme DhaT reduces 3‐HPA to 1, 3‐PDO. In the oxidation pathway, glycerol generates dihydroxyacetone (DHA) by the glycerol dehydrogenase. Then dihydroxyacetone phosphate (DHAP) is generated by the dihydroxyacetone kinase, and the pyruvate is generated through a series of enzyme reactions, accompanied by some by‐products (e.g. lactate, 2, 3‐BDO, acetate, ethanol, isobutanol and so on). These by‐products inhibit the glycerol transfer into 1, 3‐PDO, which significantly reduces the yield of 1, 3‐PDO. In addition, they are toxic to cell growth at high concentrations. In the lactate synthetic pathway, pyruvate is catalysed to lactate by l‐lactate dehydrogenase (*lldD*) (Aguilera *et al*., 2008; Fu *et al*., 2016). In the BDO synthetic pathway, (S)‐2‐acetolactate is generated by pyruvate under the catalysis of acetolactate synthase. In the next step, (S)‐2‐acetolactate is converted to acetoin under the catalysis of acetolactate decarboxylase. Finally, NADH is used as the reductant to reduce the compound to 2,3‐BDO through butanediol dehydrogenase (*budC*) (Blomqvist *et al*., 1993; Wood *et al*., 2005; Celinska and Grajek, 2009). In this study, the synthesis of lactate and BDO was investigated. Therefore, to improve the 1, 3‐PDO production by *K. pneumoniae*, overexpressing *dhaT* in the reduction pathway followed by attenuating the synthetic pathway of 2, 3‐BDO and lactate in the by‐product pathway were required in this work.

**Table 1:** Enzymes and reactions.

Enzyme	Reaction
Reduction pathway	
Glycerol dehydratase	glycerol <=> 3‐Hydroxypropanal + H_2_O
1, 3‐propanediol oxidoreductase	3‐Hydroxypropanal + NADH + H^+^ <=> Propane‐1, 3‐diol + NAD^+^
Oxidation pathway	
Glycerol dehydrogenase	glycerol + NAD+ = dihydroxyacetone + NADH + H^+^
Dihydroxyacetone Kinase	ATP + dihydroxyacetone = ADP + dihydroxyacetone phosphate
Triosephosphate isomerase	dihydroxyacetone phosphate = D‐glyceraldehyde 3‐phosphate
3‐phosphate dehydrogenase	D‐glyceraldehyde 3‐phosphate + phosphate + NAD^+^ = 3‐phospho‐D‐glyceroyl phosphate + NADH + H^+^
Phosphoglycerate kinase	ADP + 3‐phospho‐D‐glyceroyl phosphate = ATP + 3‐phospho‐D‐glycerate
2, 3‐bisphosphoglycerate‐dependent phosphoglycerate mutase	3‐phospho‐D‐glycerate = 2‐phospho‐D‐glycerate
Enolase	2‐phospho‐D‐glycerate = phosphoenolpyruvate + H_2_O
Pyruvate kinase	ADP + phosphoenolpyruvate = ATP + pyruvate
Acetyltransferase component of pyruvate dehydrogenase complex	Pyruvate=> acetyl‐CoA
Lactate dehydrogenase	pyruvate + NADH + H^+^ = (S)‐lactate + NAD^+^ pyruvate + 2 ferrocytochrome c = (S)‐lactate + 2 ferricytochrome c
Pyruvate formate‐lyase	CoA + pyruvate <=> acetyl‐CoA + formate
Acetolactate synthase	2 pyruvate = 2‐acetolactate + CO_2_
Acetolactate decarboxylase	(S)‐2‐acetolactate <=> (R)‐acetoin + CO_2_
Butanediol dehydrogenase	(S)‐acetoin + NADH + H^+^ = (2S,3S)‐butane‐2, 3‐diol + NAD^+^
Phosphate acetyltransferase	acetyl‐CoA + phosphate = CoA + acetyl phosphate
Acetate kinase	ADP + acetyl phosphate = ATP + acetate
Aldehyde dehydrogenase	acetyl‐CoA + NADH + H^+^ = acetaldehyde + CoA + NAD^+^
Alcohol Dehydrogenase	acetaldehyde + NADH + H^+^ <=> ethanol + NAD^+^
Acetyl‐CoA C‐acetyltransferase	2 acetyl‐CoA = CoA + acetoacetyl‐CoA
3‐hydroxybutyryl‐CoA dehydrogenase	3‐acetoacetyl‐CoA + NADPH + H^+^ = (S)‐3‐hydroxybutanoyl‐CoA + NADP^+^
(S)‐3‐hydroxybutanoyl‐CoA hydro‐lyase	(S)‐3‐hydroxybutanoyl‐CoA <=> crotonoyl‐CoA + H_2_O
Crotonyl‐CoA reductase	crotonoyl‐CoA + NADPH + H^+^ <=> butanoyl‐CoA + NADP^+^
Acetate CoA/acetoacetate CoA‐transferase alpha subunit	butanoyl‐CoA + acetoacetate = butanoate + acetoacetyl‐CoA
Phosphate butyryltransferase	butanoyl‐CoA + phosphate = CoA + butanoyl phosphate
Butyrate kinase	ADP + butanoylphosphate <=> ATP + butanoate
TCA	
Citrate synthase	acetyl‐CoA + H_2_O + oxaloacetate = citrate + CoA
Aconitate hydratase	citrate = cis‐aconitate + H_2_O cis‐aconitate + H_2_O = isocitrate
Isocitrate dehydrogenase	isocitrate + NADP^+^ = 2‐oxoglutarate + CO_2_ + NADPH + H^+^
2‐oxoglutarate dehydrogenase E1 component	2‐oxoglutarate + CoA + NADP^+^ <=> succinyl‐CoA + CO_2_ + NADPH + H^+^
Succinyl‐CoA synthetase	ADP + phosphate + succinyl‐CoA = ATP + succinate + CoA
Fumarate reductase	succinate + a quinone = fumarate + a quinol
Fumarate hydratase class I	fumarate + H_2_O = (S)‐malate
Malate Dehydrogenase	(S)‐malate + NAD^+^ = oxaloacetate + NADH + H^+^

Since pET28a requires induction with IPTG to start, the development of 1, 3‐PDO industrial production processes may be discouraged by expensive IPTG. Therefore, the subsequent experiments in this study were conducted without adding IPTG. The T7 promoter in pET28a was replaced by P32, hence reducing the cost of the whole process. At the same time, according to literature, P32 promoter is conducive to improving production (Minami and Shah, 2021). The fermentation experiment showed that *K.P*. pET28a‐P32‐*dhaT* was the best strain to produce 1,3‐PDO by overexpressing *dhaT*. This result may be attributed to the accumulation of 3‐HPA, an intermediate metabolite that is toxic to the strain, and detrimental to cell growth and reduces the 1, 3‐PDO production. Therefore, the higher expression of *dhaB* affected the 1, 3‐PDO production, which was consistent with the report of Zhao *et al*. (2009a,2009b) and Oh (2013). If *dhaT* was overexpressed alone, the 1, 3‐PDO production by the modified strain was only slightly higher than that of the original strain. This may be due to the fact that NADH needs to be consumed during the transformation of 3‐HPA into 1, 3‐PDO in the reduction pathway, and the overexpression of *dhaT* will lead to insufficient NADH in the reduction reaction. Thus, it is necessary to regulate the expression of some genes from the by‐product pathway, such as *lldD* and *budC* to reduce the amount of NADH required to produce by‐products, thus further increasing the production of 1, 3‐PDO.

The knockout methods were adopted in traditional methods (Kumar *et al*., 2016; Li *et al*., 2016), while the increase of 1, 3‐PDO production by knocking out of the lactate synthesis pathway was not remarkable. Direct knockout of genes in the by‐product pathway may lead to an increase in other by‐products. Additionally, completely inactivating all of the genes involved in the formation of by‐products is unreasonable due to the control of central carbon metabolic fluxes (Bro *et al*., 2006; Celinska, 2010). In fact, the diversity of by‐products reflects the delicate redox equilibrium of cells, so it is important to regulate the formation of by‐product while maintaining the overall redox equilibrium to improve glycerol utilization and 1, 3‐PDO yield. Therefore, it is necessary to consider the use of the regulatory method to reduce the generation of by‐products lactate and 2, 3‐BDO. Moreover, the regulatory method may reduce the consumption of NADH in the synthesis of lactate and 2, 3‐BDO, which will supply more NADH to DhaT in the reduction pathway, and subsequently enhance the production of 1, 3‐PDO.

*lldD* and *budC* are mainly involved in the oxidation pathway in which the by‐products (lactate and 2, 3‐BDO) during the synthesis of 1, 3‐PDO are NADH‐dependent. Thus, regulating the competitive NADH pathway can increase the concentration of NADH in the cell and reduce the concentration of the by‐product, providing NADH to the reduction pathway to ultimately improve the production of 1, 3‐PDO. Weakening expression of *budC* is beneficial to reduce the production of 2, 3‐BDO and affect the cell metabolism. By comparing the expression levels of *lldD* in L1, L2 and L3, and *budC* among B1, B2 and B3, it was found that L1 and B3 had the lowest expression levels, indicating that the regulatory effects of these two sgRNAs were the best. As a result, the combination of L1 and B3 might perform better. The results showed that the production of 1, 3‐PDO by *K.P*. L1‐B3‐pRH2521 was higher than that of the original strain. Compared with the complete deletion of lactate and the whole 2, 3‐BDO pathway would not inhibit GDHt because of the massive carbon metabolism of pyruvate nodes, thus affecting the production of 1, 3‐PDO (Kumar *et al*., 2016).

By combining overexpression and regulation with CRISPR‐dCas9, it could be found that the 1, 3‐PDO production in the presence of *K.P*. L1‐B3‐pRH2521‐P32‐*dhaT* was the highest (57.85 g l^−1^), suggesting that the inhibiting the expression of *lldD* and *budC* in pathway of lactate and 2, 3‐BDO might reduce the consumption of NADH and enhance the conversion of 3‐HPA to 1, 3‐PDO.

Simultaneous multiple gene modification is more beneficial to 1,3‐PDO production than traditional single‐gene modification methods (e.g., single overexpression (Zheng *et al*., 2006; Hao *et al*., 2008; Zhao *et al*., 2009a,2009b; Ma *et al*., 2010; Zhu *et al*., 2015), knockout (Horng *et al*., 2010; Guo *et al*., 2013), and regulation (Wei *et al*., 2014; Hirokawa *et al*., 2017; Lee *et al*., 2018, 2019).

In summary, this is the first time the *dhaT* has been overexpressed in *K. pneumoniae*, and the expression of *lldD* and *budC* was simultaneously inhibited to enhance the performance of the strain in the CRISPR‐dCas9 system. This method is relatively simple and efficient and only requires to transfer two plasmids into bacteria simultaneously to achieve the effects of the overexpression and attenuation. Compared with the original (45.86 g l^−1^), the production of 1, 3‐PDO by *K.P*. L1‐B3‐pRH2521‐P32‐*dhaT* was successfully increased up to 57.85 g l^−1^. In *K. pneumoniae*, the overexpression and inhibition of the gene expression not only does not affect the NADH balance in the metabolic process, but also enhancing the final 1, 3‐PDO production. Therefore, this method may have certain advantages over the NADH imbalance in metabolic process compared with the traditional knockout, and finally decreases the yield of target products. There are many genes in *K. pneumoniae* that need to be further studied, such as *dhaD*, *dhaK*, *ldhA*, *budA*, *ldhA*, *poxB*, *pta*, *ackA*, *gltA*, *arcA* and so on. These genes can be overexpressed and regulated in combination to promote the production of 1, 3‐PDO by CRISPR‐dCas9. These findings provide a reference for the direction of the genetic modification for the target products to promote the productive ability of other strains.

## Experimental procedures

### Strains, microorganisms and cultivations

*K. pneumoniae* ATCC 15380 was purchased from American Type Culture Collection (ATCC, Manassas, VA, USA). The microorganisms and cultivations were provided in the Appendix S1.

### Analytical methods and instrumentation

The concentrations of 1, 3‐PDO, glycerol and 2, 3‐BDO were measured with an HPX‐87H column (300 × 7.8 mm^2^) (Bio‐Rad, Palo AHO, CA, USA) with a differential refractive index detector (SFD GmbH, Schambeck, Germany) (as shown in Fig. 2C). 5 mM aqueous H_2_SO_4_ solution was used as a mobile phase with a flow rate of 0.5 ml min^−1^ at a working temperature at 65°C.

The concentration of lactate was measured with an MP C18 column (250 mm × 4.6 mm, 5 µm; Agilent) by a liquid chromatography on a 1100 series instrument (Agilent, Santa Clara, CA, USA) (as shown in Fig. 3D). 5 mM H_2_SO_4_ (with 5% acetonitrile) solution was used as a mobile phase with a flow rate of 0.5 ml min^−1^ and a working temperature at 35°C.

### Plasmids, primers and sequences

The plasmids used in this study are listed in Table 2. The primers used for gene deletion, complementation and overexpression are shown in Table 3. The sequences of the devices and gene fragments are shown in Table 4.

**Table 2:** Strains and plasmids used in this study.

Strain/Plasmids	Descriptions	Source
*K. pneumoniae*		
15380	Mutant derived from ATCC 15380	USA
P32‐*dhaB*	P32‐*dhaB*, Kan^R^	This study
P32‐*dhaT*	P32‐*dhaT*, Kan^R^	This study
P32‐*dhaB*‐*dhaT*	P32‐*dhaB*‐*dhaT*, Kan^R^	This study
L1	*lldD* sgRNA‐L1, HmB^R^, Kan^R^	This study
L2	*lldD* sgRNA‐L2, HmB^R^, Kan^R^	This study
L3	*lldD* sgRNA‐L3, HmB^R^, Kan^R^	This study
B1	*budC* sgRNA‐B1, HmB^R^, Kan^R^	This study
B2	*budC* sgRNA‐B2, HmB^R^, Kan^R^	This study
B3	*budC* sgRNA‐B3, HmB^R^, Kan^R^	This study
L1‐B3‐pRH2521‐P32‐*dhaT*	*lldD* sgRNA‐L1, *budC* sgRNA‐B3	This study
*E.coli*		
DH5α	Plasmid construction and general cloning	Novagen, USA
Plasmids		
pET‐28a	Vector for protein expression, Kan^R^	Novagen, USA
pET‐P32	P32 instead of T7 promoter Kan^R^	This study
pET‐P32‐*dhaB*	*dhaB* expression vector based on pET‐28a(+), Kan^R^	This study
pET‐P32‐*dhaT*	*dhaT* expression vector based on pET‐28a(+), Kan^R^	This study
pET‐P32‐*dhaB*‐*dhaT*	*dhaB* and *dhaT* expression vector based on pET‐28a(+), Kan^R^	This study
pRH2521	Expression of sgRNA from a imyc promoter (Pimyc), HmB^R^	Addgene, USA
pRH2502	Expression of dcas9 D10A H840A from a TetR‐regulated uvtetO promoter, Kan^R^	Addgene, USA
L1‐pRH2521	L1‐sgRNA	This study
L2‐pRH2521	L2‐sgRNA	This study
L3‐pRH2521	L3‐sgRNA	This study
B1‐pRH2521	B1‐sgRNA	This study
B2‐pRH2521	B2‐sgRNA	This study
B3‐pRH2521	B3‐sgRNA	This study
L1‐B3‐pRH2521	L1‐sgRNA, B3‐sgRNA	This study

**Table 3:** Primers used for gene deletion, complementation, and overexpression.

Primers	Sequence (5’–3’)
p32‐F	TAAACAAAATTATTTCTAGATCGAATTCGGTCCTCGGGATAT
p32‐R	TTTTGATCTTTTCATTTGTATTCCCTATTCAAAATTCCTCCGAATATTTTTTTACCTA
*dhaB*‐F	CGGAGGAATTTTGAATAGGGAATACAAATGAAAAGATCAAAACGATTTGCAGTACT
*dhaB*‐R1	GGCGTAGAGGATCGAGATCTTTAGCTTCCTTTACGCAGCTTATG
*dhaB*‐R2	TGTGTATCGTCGCCATTTGTATTCCCTAGCTGACCTCCGCTTAGCTT
*dhaT*‐F1	CGGAGGAATTTTGAATAGGGAATACAAATGGCGACGATACACAGCACAAT
*dhaT*‐F2	TAAGCGGAGGTCAGCTAGGGAATACAAATGGCGACGATACACAGCACAAT
*dhaT*‐R	GGCGTAGAGGATCGAGATCTTTAGGCATGTTCTGGATACAGC
p32‐*dhaB*‐*dhaT*‐F	CGGCCGCAAGCTTGTCGA
p32‐*dhaB*‐*dhaT*‐R	AGTAGTAGGTTGAGGCCGTTGAGCA
pet28‐p32‐GFP‐F	TAAACAAAATTATTTCTAGATCGAATTCGGTCCTCGGGATAT
pet28‐p32‐GFP‐R	GGCGTAGAGGATCGAGATCTCTAGAACTGGCATGCATCTTTGTA
pBluescriptKS	TCGAGGTCGACGGTATC
pBR322ori‐F	GGGAAACGCCTGGTATCTTT
EBV‐rev	GTGGTTTGTCCAAACTCATC
2521‐F	TATTGGATCGTCGGCACCGTC
2521‐R	CTGATCATCTGCGGCTTGGAG
lldDsgRNA‐L1‐F	GGGAGACTCAGCCCTCCTCTCCTGG
lldDsgRNA‐L1‐R	AAACCCAGGAGAGGAGGGCTGAGTC
lldDsgRNA‐L2‐F	GGGAGCAATAATTTCATCCATCCCC
lldDsgRNA‐L2‐R	AAACGGGGATGGATGAAATTATTG
lldDsgRNA‐L3‐F	GGGAGAATTTTACCTTCGGTGGGAT
lldDsgRNA‐L3‐R	AAACATCCCACCGAAGGTAAAATT
budCsgRNA‐B1‐F	GGGAGTTTCTTATATTTGTTGAACG
budCsgRNA‐B1‐R	AAACCGTTCAACAAATATAAGAAA
budCsgRNA‐B2‐F	GGGAGTTGGAACTGTGAGCTGAATC
budCsgRNA‐B2‐R	AAACGATTCAGCTCACAGTTCCAA
budCsgRNA‐B3‐F	GGGAGAACCAGCATGGTTTCTATAT
budCsgRNA‐B3‐R	AAACATATAGAAACCATGCTGGTTC
Ptet‐B3‐sgRNA‐F	ACGAGTATGCATGATCTGTGCGTTCGCAC
Ptet‐B3‐sgRNA‐R	TGACTCGCTAGCTGCATATTAATTAAATCGATAAAAAAGCAC

**Table 4:** Sequences of devices and gene fragments.

Fragment	Sequence
P32	TCGAATTCGGTCCTCGGGATATGATAAGATTAATAGTTTTAGCTATTAATCTTTTTTTATTTTTATTTAAGAATGGCTTAATAAAGCGGTTACTTTGGATTTTTGTGAGCTTGGACTAGAAAAAAACTTCACAAAATGCTATACTAGGTAGGTAAAAAAATATTCGGAGGAATTTTGAA
RBS	TAGGGAATACAA
*dhaB*	ATGAAAAGATCAAAACGATTTGCAGTACTGGCCCAGCGCCCCGTCAATCAGGACGGGCTGATTGGCGAGTGGCCTGAAGAGGGGCTGATCGCCATGGAC>AGCCCCTTTGACCCGGTCTCTTCAGTAAAAGTGGACAACGGTCTGATCGTCGAGCTGGACGGCAAACGCCGGGACCAGTTTGACATGATCGACCGATTTATCGCCGATTACGCGATCAACGTTGAGCGCACAGAGCAGGCAATGCGCCTGGAGGCGGTGGAAATAGCCCGCATGCTGGTGGATATTCACGTCAGTCGGGAGGAGATCATTGCCATCACTACCGCCATCACGCCGGCCAAAGCGGTCGAGGTGATGGCGCAGATGAACGTGGTGGAGATGATGATGGCGCTGCAGAAGATGCGTGCCCGCCGGACCCCCTCCAACCAGTGCCACGTCACCAATCTCAAAGATAATCCGGTGCAGATTGCTGCTGACGCCGCCGAGGCCGGGATCCGCGGCTTCTCAGAACAGGAGACCACGGTCGGTATCGCGCGCTATGCGCCGTTTAACGCCCTGGCGCTGTTGGTCGGTTCGCAGTGCGGCCGCCCCGGCGTTTTGACGCAGTGCTCGGTGGAAGAGGCCACCGAGCTGGAGCTGGGCATGCGTGGCTTAACCAGCTACGCCGAGACGGTGTCGGTCTACGGCACCGAAGCGGTATTTACCGACGGCGATGATACTCCGTGGTCAAAGGCGTTCCTCGCCTCGGCCTACGCCTCCCGCGGGTTGAAAATGCGCTACACCTCCGGCACCGGATCCGAAGCGCTGATGGGCTATTCGGAGAGCAAGTCGATGCTCTACCTCGAATCGCGCTGCATCTTCATTACCAAAGGCGCCGGGGTTCAGGGGCTGCAAAACGGCGCGGTGAGCTGTATCGGCATGACCGGCGCTGTGCCGTCGGGCATTCGGGCGGTGCTGGCGGAAAACCTGATCGCCTCTATGCTCGACCTCGAAGTGGCGTCCGCCAACGACCAGACTTTCTCCCACTCGGATATTCGCCGCACCGCGCGCACCCTGATGCAGATGCTGCCGGGCACCGACTTTATTTTCTCCGGCTACAGCGCGGTGCCGAACTACGACAACATGTTCGCCGGCTCGAACTTCGATGCGGAAGATTTTGATGATTACAACATCCTGCAGCGTGACCTGATGGTTGACGGCGGCCTGCGTCCGGTGACCGAGGCGGAAACCATTGCCATTCGCCAGAAAGCGGCGCGGGCGATCCAGGCGGTTTTCCGCGAGCTGGGGCTGCCGCCAATCGCCGACGAGGAGGTGGAGGCCGCCACCTACGCGCACGGTAGCAACGAGATGCCGCCGCGTAACGTGGTGGAGGATCTGAGTGCGGTGGAAGAGATGATGAAGCGCAACATCACCGGCCTCGATATTGTCGGCGCGCTGAGCCGCAGCGGCTTTGAGGATATCGCCAGCAATATTCTCAATATGCTGCGCCAGCGGGTCACCGGCGATTACCTGCAGACCTCGGCCATTCTCGATCGGCAGTTCGAGGTGGTGAGTGCGGTCAACGACATCAATGACTATCAGGGGCCGGGCACCGGCTATCGCATCTCTGCCGAACGCTGGGCGGAGATCAAAAATATTCCGGGCGTGGTTCAGCCCGACACCATTGAATAA
RBS	TAGGGAATACAA
*dhaT*	TGGCGACGATACACAGCACAATCATCAGCGGGGCTGGCGCCTCATCGGCCCTGCTCCCGCTGCTGGCCGGTAAAACCTCAATCCTGCTGGTGACCGACAAGAACGTCGGGGCGCTGGAGGCAACCCAGGCGATTCATCGCCTGCTGGCGGCTGAAGGGCGTGAAGTTGAGATCATTGACAGCGTGCCAGCTGAGCCCAACCATCACGATGTTACGCAGATCGTCAGCCAGCTGGGCGCCAGCCAGCCGCAGATGGTCGTTGGTATCGGCGGGGGGAGCGTACTGGATGTAGCAAAACTGCTGTCAGTACTTCTGCACCCTGAGGCGCCCTCGCTGACGTCCCTGCTGGCAGGCGAGCAGCCGCAACGACGAATTTGCTCATTGTTAATCCCGGCCACCGCGGGCACCGGATCCGAAGCGACGCCGAACGCCATTCTGGCGATCCCCGAGCAGCAGACGAAGGTGGGGATCATCTCCCCGGTGCTGCTTCCGGACTATGTCGCCCTGCTGCCGGAGCTGACCACCAGCATGCCGCCCAGCATCGCGGCGTCGACCGGAATCGATGCTCTGTGTCATCTGCTGGAGTGCTTTACCTCCACCGTCGCCAACCCGGTGAGCGATAACGCCGCGTTAATCGTTTACACAAGCTGGTACGCCATATCGAGCGTTCGGTAAATCAGCCGCAGGATCTGACGGCGAAGCTGGAGATGCTCTGGGCCTCGTGGTACGGCGGCGCAGCGATCAACTATGCCGGCACTCACCTGGTGCATGCGCTCTCCTATCCGCTTGGCGGTACCTGGCACCTGCCGCACGGGGTGGCCAACGCCATTCTGCTGGCGCCCTGCATGCGAGTGGTCCGCCCTCACGCGGTGGCGAAGTTCGCTCAAGTCTGGGACCTGATCCCCGATGCAGATCGCACGCTCAGCGCGGAAGAGAAATCCCATGCGCTGGTCGCATGGCTGGCGGCGCTGGTCAAACGACTGCCGCTGCCGGACAACCTGGCCGCGCTAGGCGTGCCTCAGGAGAGTATTTCCGCCCTCAGCGCAGCGGCGCAAAACGTCAAACGCCTGATGAACAATGCCCCATGCAGCGTCAGCCGCGAAGAGATCGCGGCCATCTACCAAACGCTGTATCCAGAACATGCCTAA
*lldD*	TTACGCGGCGTTATTCTGCTTCAGCGCATCGAAGGTCTGCAGCGCTTCGGCGTTCTGCACCAGCGAATCACGGCTGATTTCACGGATACTTTTCGCCCCGGTGAGGGTCATCGCCACTTTCATCTCTTTCTCGATGAGGTTCAGCAGATTCGCCACGCCCTGCTTACCGTGGGTCGCCAGGGCGTACAGGTAAGCGCGGCCCAGTAGCACGCTGTCGGCGCCCAGAGCGATCATCCGCACCACGTCGAGCCCGTTACGGATGCCGCTGTCGGCGAGAATAGTGATGTCGCCTTTCACCGCGTCGGCAATTGCCGGCAGGGCGCGGGCCGAGGAGAGCACGCCATCGAGCTGGCGGCCGCCGTGGTTGGAGACGACAATGCCGTCAGCGCCGAAGCGCACCGCGTCGCGGGCGTCCTCCGGATCGAGGATCCCTTTGATCACCATCGGACCGTCCCAGAAATCGCGGATCCACTCCAGGTCTTTCCATGAAATCGACGGATCGAAGTTATTCGCCAGCCAGCCAATGTAATCCTCAAGCCCGGTCGGTTTGCCGAGATAGGCGGAGATATTGCCCAGATCGTGCGGGCGACCGTTAAGGCCGACGTCCCACGCCCACTGCGGATGGGTGACGGCCTGCCAGTAGCGGCGCAGGGCGGCGTTCGGGCCGCTCATGCCTGAATGGGCGTCGCGGTAGCGGGCGCCGGGAGTCGGCATATCGACGGTAAAGACCAGCGTCGAGCAGCCGGCGGCTTTGGCGCGCTCCAGCGCATTGCGCATAAAGCCGCGGTCGCGCAGGACATACAGCTGGAACCACATCGGGCGCTTGATGGTCGGGGCGACCTCTTCGATCGGGCAGACGGAAACGGTAGAGAGGGTGAACGGAATGCCTTTGTCGTCCGCCGCGCCGGCGGCCTGCACTTCGCCGCGGCGGGCGTACATCCCGCACAGCCCGACCGGCGCCAACGCCGTCGGCATCGCCAGCTTCTCGTTGAATAATGTCGTTTCCAGACTCAGGTCCGACATATTGCGCAAAATACGCTGGCGCAAGGCTACATCGGAAAGGTCCTCGACGTTGCGGCGCAGGGTATGTTCGGCGTAGGCGCCGCCGTCAATATAGTGGAACAGAAACGGCGGCAGGATACGCTGTGCGGCGGCGCGATAGTCGCTGGCGGCGGAAATAATCAT
*budC*	TTAGTTAAACACCATGCCGCCGTCGATCAGCAATGACTGACCGGTCATATAATCAGAATCCGGGCTGGCAAGATAGGAGACGCAGGCGGCGACATCTTCCGGCTCGGACAGGCGGCCGAGGGTGATGCGTTTGGCGAACTCGGCGGTACCGTAGCCCAGCGGTTTACCGGCGGCTTCGGACACCTGGCGGTCAATTTCGGCCCACATCGGCGTTTTGACAATCCCCGGGCAGTAGCCGTTGACCGTGATGCCCAGCGGCGCGAGGTCGCGAGCGGCGGTCTGGGTTAAGCCGCGTACCGCGAATTTACTCGAGCTATATACCGCCAGCTCCGGGTTGCCGACGTGGCCGGCCTGGGAACAGGCGTTGATGATTTTCCCGCCGTGACCCTCTTTCTTAAAGGCCTCGACCGCTGCCTGGATGCCCCAGATCACCCCTTTGACGTTGATGTTGTAGACTTTGTCGACAATCTCCGGGGTAATGGACTCGATCGGCGTGGATGGCGCCACGCCGGCGTTGTTGACGATGACGTCGAAGCCGCCCAGCGTTTTGCGCGCCTGTTCGACGGCGGCAAATACCTGGTCGCGGTCAGAAACATCCACTTTCACCGCCATGGCGCGGCCGCCGGCCTGGTTGATTTCGGAGGCGACCGCTTTGGCGGTGGCGTCGTTATAATCGGCAATGGCCACGGCAAATCCATCCTTCACCAGACGAAGGGCGATAGCTTTACCAATCCCCTGGCCGGCGCCGGTAACAAGTGCGACTTTTTTCAT

### Construction of pET28a‐P32‐*dhaB*, pET28a‐P32‐*dhaT*, pET28a‐P32‐*dhaB*‐*dhaT*

**Fig. 5:**
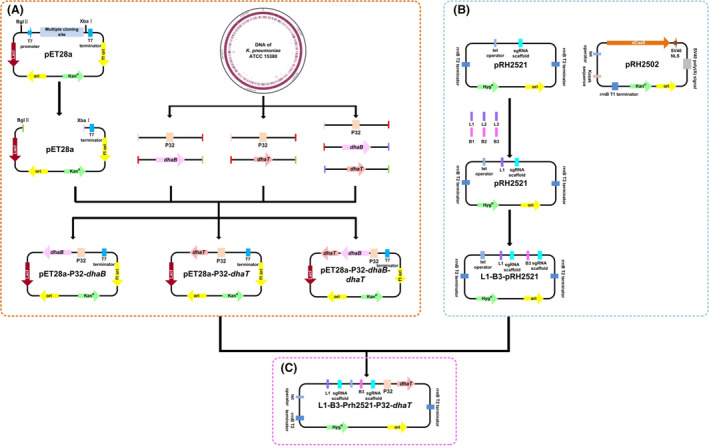
Schematic representation of the strategies used for (A) construction of pET28a‐*dhaB*, pET28a‐*dhaT* and pET28a‐*dhaB*‐*dhaT*. B. Construction of L1‐pRH2521, L2‐pRH2521, L3‐pRH2521, B1‐pRH2521, B2‐pRH2521, B3‐pRH2521 and L1‐B3‐pRH2521. C. Construction of *dhaT*‐L1‐B3‐pRH2521.

The P32 promoter was known from the literature reports (Van der Vossen *et al*., 1987), and the sequences of P32 promoter was synthesized by Tsingke Biotechnology Co., Ltd. All plasmids were constructed in *Escherichia coli* DH 5α. Total DNA was extracted from *K. pneumoniae* by DNA extraction kit (Tiangen, Beijing, China). Genomic DNA of *K. pneumoniae* NCTC418 (National Center for Biotechnology Information (NCBI) reference sequence: NZ_LR134213.1) was used as a template for polymerase chain reaction (PCR) to amplify *dhaB* and *dhaT* gene clusters. The sequence of *dhaB* was amplified with *dhaB*‐F and *dhaB*‐R primers. The pET28a was digested with XbaⅠ and BglⅡ to obtain the linearized plasmid (as shown in Fig. 5A), and then pET28a, P32 and *dhaB* were connected using a BM seamless cloning kit (Biomed, Beijing, China) to form pET28a‐P32‐*dhaB*. The construction of pET28a‐P32‐*dhaT* and pET28a‐P32‐*dhaB*‐*dhaT* was similar to that of pET28a‐P32‐*dhaB*, and the construction process is shown in Fig. 5A.

### Construction of L1‐pRH2521, L2‐pRH2521, L3‐pRH2521, B1‐pRH2521, B2‐pRH2521, B3‐pRH2521 and L1‐B3‐pRH2521

As described previously (Jha *et al*., 2020), a CRISPRi‐based approach was used to regulate the strains. Single‐guide RNA (sgRNA) targeting *lldD* and *budC* at site L1~L3 and B1~B3 (as shown in Fig. 5B) were cloned into pRH2521 plasmid at the BbsI site. Three candidate target sites were selected for each target gene. The L1‐pRH2521, L2‐pRH2521, L3‐pRH2521, B1‐pRH2521, B2‐pRH2521 and B3‐pRH2521 were constructed (as shown in Fig. 5B). The sgRNA was designed by Benchling (https://www.benchling.com).

Ptet‐B3‐sgRNA PCR was recovered by PCR with primers Ptet‐B3‐sgRNA‐F and Ptet‐B3‐sgRNA‐R using B3‐pRH2521 as the template. The inserted fragment was recovered by double enzyme digestion with NsiI and NheI. The L1‐pRH2521 was digested back with NsiI and NheI to obtain the vector. The fragment was connected with the vector to obtain L1‐B3‐pRH2521 (as shown in Fig. 5B).

### Construction of L1‐B3‐pRH2521‐P32‐*dhaT*

In plasmid pET28a‐P32‐*dhaT*, P32‐*dhaT* was cut off with NheI and MluI (4889 bp), which was recycled as fragments by gel cutting. L1‐B3‐pRH2521 was recovered by NheI and MluI double enzyme digestion to obtain the vector. The fragment was connected to the carrier. Finally, the plasmid L1‐B3‐pRH2521‐P32‐*dhaT* was obtained (as shown in Fig. 5C).

### Transformation and screening

Take plasmid pRH2521 as an example. The constructed plasmid pRH2521 was transformed into *E. coli* DH5α. Colonies were selected on LB plates containing hygromycin (50 µg ml^−1^). PCR and sequencing were used to verify the accuracy of plasmid. Finally, plasmid pRH2521 was extracted from *E. coli* DH5α using the plasmid extraction kit (Biomed). Then, the extracted pRH2521 and pRH2502 (kanamycin resistant to dCas9 expression) were electrically transferred into *K. pneumoniae*, respectively, and colonies were selected on LB plates containing hygromycin (50 µg ml^−1^) and kanamycin (25 µg ml^−1^). Additionally, the plasmids were transferred to *K. pneumoniae* by electroporation to verify the accuracy of plasmids. Then, the plates were incubated at 37°C for 12–24 h. The stability of the plasmid was the ratio of the percentage of colonies on the antibiotic agar plates over those on the plates without antibiotics. To induce sgRNA and dCas9 expression, anhydrotetracycline was added to the cultures to achieve a final concentration of 200 ng ml^−1^ at 6 h (Jha *et al*., 2020). The plasmid pET28a (kanamycin 50 µg ml^−1^) was also constructed as described above. The recombinant plasmids were transformed into *E. coli* to obtain transformants and transformed into *K. pneumoniae* by electroporation.

### Quantitative Real‐Time PCR (qRT‐PCR) analysis

The transcriptional levels of *budC* and *lldD* in the K.P. ATCC15380, L1~L3 and B1~B3 were measured by qRT‐PCR. The total RNA of the bacteria was extracted by using the RNA prepPure culture bacterial total RNA extraction kit (Tiangen) according to the operation instructions, and treated with DNase I to remove the residual DNA fragments. The RNA samples were quantified by spectrophotometer at 260 and 280 nm, and purified by 1% formaldehyde agarose gel electrophoresis. cDNA synthesis was completed by using the fast quant cDNA first strand synthesis kit (Tiangen) according to the operation instructions. The PCR reaction was designed according to the *K. pneumoniae* NCTC 418 genomic sequence at GenBank in NCBI database. The synthetic primers of the target gene were designed by primer design software (Primer Premier 5.0). qRT‐PCR was performed with the Real Master Mix (SYBRGreen) kit (Tiangen) according to the operation instructions using the *K. pneumoniae* 16S rRNA gene as the internal standard control.

Relative quantitation 2^‐ΔΔCT^ method was used to analyse the qRT‐PCR data. To calculate the *C*_T_ value obtained from the experiment, and the relative differences of gene transcription were obtained (Wang *et al*., 2021a,2021b).

## Consent for publication

All authors consent to publish this manuscript.

## Author’s contributions

XW completed the experiment, analysed the data and wrote the manuscript. SXL, YY and PW participated in the manuscript revise. YCL, and LZ participated in some experiments. JWX and WSC helped to edit the manuscript and involved in discussion in the manuscript preparation. All authors read and approved the final manuscript.

## Supporting information


**Appendix S1**. The microorganism and cultivations.

## References

[mbt214033-bib-0001] Aguilera, L. , Campos, E. , Gimenez, R. , Badia, J. , Aguilar, J. , and Baldoma, L. (2008) Dual role of LldR in regulation of the lldPRD operon, involved in L‐lactate metabolism in *Escherichia coli* . J Bacteriol 190: 2997–3005. 18263722 10.1128/JB.02013-07 PMC2293229

[mbt214033-bib-0002] Avci, F.G. , Huccetogullari, D. , and Azbar, N. (2014) The effects of cell recycling on the production of 1,3‐propanediol by *Klebsiella pneumoniae* . Bioprocess Biosyst Eng 37: 513–519. 23892658 10.1007/s00449-013-1018-z

[mbt214033-bib-0003] Blomqvist, K. , Nikkola, M. , Lehtovaara, P. , Suihko, M.L. , Airaksinen, U. , Stråby, K.B. , *et al*. (1993) characterization of the genes of the 2,3‐butanediol operons from *Klebsiella‐terrigena* and *Enterobacter‐aerogenes* . J Bacteriol 175: 1392–1404. 8444801 10.1128/jb.175.5.1392-1404.1993 PMC193226

[mbt214033-bib-0004] Bro, C. , Regenberg, B. , Forster, J. , and Nielsen, J. (2006) In silico aided metabolic engineering of *Saccharomyces* cerevisiae for improved bioethanol production. Metab Eng 8: 102–111. 16289778 10.1016/j.ymben.2005.09.007

[mbt214033-bib-0005] Casali, S. , Gungormusler, M. , Bertin, L. , Fava, F. , and Azbar, N. (2012) Development of a biofilm technology for the production of 1,3‐propanediol (1,3‐PDO) from crude glycerol. Biochem Eng J 64: 84–90.

[mbt214033-bib-0006] Celinska, E. (2010) Debottlenecking the 1,3‐propanediol pathway by metabolic engineering. Biotechnol Adv 28: 519–530. 20362657 10.1016/j.biotechadv.2010.03.003

[mbt214033-bib-0007] Celinska, E. , and Grajek, W. (2009) Biotechnological production of 2,3‐butanediol‐Current state and prospects. Biotechnol Adv 27: 715–725. 19442714 10.1016/j.biotechadv.2009.05.002

[mbt214033-bib-0008] Chen, H.W. , Fang, B.S. , and Hu, Z.D. (2005) Optimization of process parameters for key enzymes accumulation of 1,3‐propanediol pro uction from *Klebsiella pneumoniae* . Biochem Eng J 25: 47–53.

[mbt214033-bib-0009] Chen, W.‐C. , Chuang, C.‐J. , Chang, J.‐S. , Wang, L.‐F. , Soo, P.‐C. , Wu, H.‐S. , *et al*. (2020) Exploring dual‐substrate cultivation strategy of 1,3‐propanediol production using *Klebsiella pneumoniae* . Appl Biochem Biotechnol 191: 346–359. 31863348 10.1007/s12010-019-03208-6

[mbt214033-bib-0010] Cheng, K.K. , Liu, D.H. , Sun, Y. , and Liu, W.B. (2004) 1,3‐propanediol production by *Klebsiella pneumoniae* under different aeration strategies. Biotech Lett 26: 911–915. 10.1023/b:bile.0000025902.44458.f4 15269540

[mbt214033-bib-0011] Cheng, K.K. , Zhang, J.A. , Liu, D.H. , Sun, Y. , Yang, M.D. , and Xu, J.M. (2006) Production of 1,3‐propanediol by *Klebsiella pneumoniae* from glycerol broth. Biotech Lett 28: 1817–1821. 10.1007/s10529-006-9158-8 16912919

[mbt214033-bib-0012] Deaner, M. , Holzman, A. , and Alper, H.S. (2018) Modular ligation extension of guide RNA operons (LEGO) for multiplexed dCas9 regulation of metabolic pathways in *Saccharomyces cerevisiae* . Biotechnol J 13: 1–11. 10.1002/biot.201700582 29663663

[mbt214033-bib-0013] Fu, J. , Huo, G. , Feng, L. , Mao, Y. , Wang, Z. , Ma, H. , *et al*. (2016) Metabolic engineering of Bacillus subtilis for chiral pure meso‐2,3‐butanediol production. Biotechnol Biofuels 9: 1–14. 27099629 10.1186/s13068-016-0502-5 PMC4837526

[mbt214033-bib-0014] Gonzalez, R. , Murarka, A. , Dharmadi, Y. , and Yazdani, S.S. (2008) A new model for the anaerobic fermentation of glycerol in enteric bacteria: trunk and auxiliary pathways in *Escherichia coli* . Metab Eng 10: 234–245. 18632294 10.1016/j.ymben.2008.05.001

[mbt214033-bib-0015] Guo, X.K. , Fang, H.Y. , Zhuge, B. , Zong, H. , Song, J. , Zhuge, J. , and Du, X.X. (2013) budC knockout in *Klebsiella pneumoniae* for bioconversion from glycerol to 1,3‐propanediol. Biotechnol Appl Biochem 60: 557–563. 23586646 10.1002/bab.1114

[mbt214033-bib-0016] Hao, J. , Wang, W. , Tian, J.S. , Li, J.L. , and Liu, D.H. (2008) Decrease of 3‐hydroxypropionaldehyde accumulation in 1,3‐propanediol production by over‐expressing dhaT gene in *Klebsiella pneumoniae* TUAC01. J Ind Microbiol Biotechnol 35: 735–741. 18365261 10.1007/s10295-008-0340-y

[mbt214033-bib-0017] Hirokawa, Y. , Maki, Y. , and Hanai, T. (2017) Improvement of 1,3‐propanediol production using an engineered cyanobacterium, *Synechococcus elongatus* by optimization of the gene expression level of a synthetic metabolic pathway and production conditions. Metab Eng 39: 192–199. 27998670 10.1016/j.ymben.2016.12.001

[mbt214033-bib-0018] Horng, Y.T. , Chang, K.C. , Chou, T.C. , Yu, C.J. , Chien, C.C. , Wei, Y.H. , and Soo, P.C. (2010) Inactivation of dhaD and dhaK abolishes by‐product accumulation during 1,3‐propanediol production in *Klebsiella pneumoniae* . J Ind Microbiol Biotechnol 37: 707–716. 20379761 10.1007/s10295-010-0714-9

[mbt214033-bib-0019] Jha, R.K. , Udupa, S. , Rai, A.K. , Rani, P. , Singh, P.R. , Govind, S. , and Nagaraja, V. (2020) Conditional down‐regulation of GreA impacts expression of rRNA and transcription factors, affecting *Mycobacterium smegmatis* survival. Sci Rep 10. 10.1038/s41598-020-62703-7 PMC7118132 32242064

[mbt214033-bib-0020] Ju, J.‐H. , Heo, S.‐Y. , Choi, S.‐W. , Kim, Y.‐M. , Kim, M.‐S. , Kim, C.‐H. , and Oh, B.‐R. (2021) Effective bioconversion of 1,3‐propanediol from biodiesel‐derived crude glycerol using organic acid resistance–enhanced Lactobacillus reuteri JH83. Bioresour Technol 337: 125361. 34320778 10.1016/j.biortech.2021.125361

[mbt214033-bib-0021] Ju, J.H. , Jeon, S.G. , Lee, K.M. , Heo, S.Y. , Kim, M.S. , Kim, C.H. , and Oh, B.R. (2021) The biocatalytic production of 3‐hydroxypropionaldehyde and evaluation of its stability. Catalysts 11: 1139.

[mbt214033-bib-0022] Kumar, V. , Durgapal, M. , Sankaranarayanan, M. , Somasundar, A. , Rathnasingh, C. , Song, H.H. , *et al*. (2016) Effects of mutation of 2,3‐butanediol formation pathway on glycerol metabolism and 1,3‐propanediol production by *Klebsiella pneumoniae* J2B. Biores Technol 214: 432–440. 10.1016/j.biortech.2016.04.032 27160953

[mbt214033-bib-0023] Kumar, V. , and Park, S. (2018) Potential and limitations of *Klebsiella pneumoniae* as a microbial cell factory utilizing glycerol as the carbon source. Biotechnol Adv 36: 150–167. 29056473 10.1016/j.biotechadv.2017.10.004

[mbt214033-bib-0024] Lama, S. , Seol, E. , and Park, S. (2017) Metabolic engineering of *Klebsiella pneumoniae* J2B for the production of 1,3‐propanediol from glucose. Biores Technol 245: 1542–1550. 10.1016/j.biortech.2017.05.052 28549809

[mbt214033-bib-0025] Lama, S. , Seol, E. , and Park, S. (2020) Development of *Klebsiella pneumoniae* J2B as microbial cell factory for the production of 1,3‐propanediol from glucose. Metab Eng 62: 116–125. 32898717 10.1016/j.ymben.2020.09.001

[mbt214033-bib-0026] Lee, J.H. , Jung, H.M. , Jung, M.Y. , and Oh, M.K. (2019) Effects of gltA and arcA Mutations on Biomass and 1,3‐Propanediol Production in *Klebsiella pneumoniae* . Biotechnol Bioprocess Eng 24: 95–102.

[mbt214033-bib-0027] Lee, J.H. , Jung, M.‐Y. , and Oh, M.‐K. (2018) High‐yield production of 1,3‐propanediol from glycerol by metabolically engineered *Klebsiella pneumoniae* . Biotechnol Biofuels 11: 1–13. 29657579 10.1186/s13068-018-1100-5 PMC5890353

[mbt214033-bib-0028] Li, L.L. , Zhou, S. , Ji, H.S. , Gao, R. , and Qin, Q.W. (2014) Optimization of fermentation conditions for 1,3‐propanediol production by marine *Klebsiella pneumonia* HSL4 using response surface methodology. Chin J Oceanol Limnol 32: 1036–1045.

[mbt214033-bib-0029] Li, Y. , Li, S. , Ge, X.Z. , and Tian, P.F. (2016) Development of a Red recombinase system and antisense RNA technology in *Klebsiella pneumoniae* for the production of chemicals. Rsc Advances 6: 79920–79927.

[mbt214033-bib-0030] Liu, H.Y. , Ni, J. , Xu, P. , and Tao, F. (2018) Enhancing light‐driven 1,3‐propanediol production by using natural compartmentalization of differentiated cells. Acs Synth Biol 7: 2436–2446. 30234972 10.1021/acssynbio.8b00239

[mbt214033-bib-0031] Ma, Z. , Rao, Z.M. , Zhuge, B. , Fang, H.Y. , Liao, X.R. , and Zhuge, J. (2010) Construction of a novel expression system in *Klebsiella pneumoniae* and its application for 1,3‐propanediol production. Appl Biochem Biotechnol 162: 399–407. 19728170 10.1007/s12010-009-8743-4

[mbt214033-bib-0032] Minami, S.A. , and Shah, P.S. (2021) Transient light‐activated gene expression in Chinese hamster ovary cells. BMC Biotechnol 21: 1–8. 33541329 10.1186/s12896-021-00670-1 PMC7863527

[mbt214033-bib-0033] Nodvig, C.S. , Hoof, J.B. , Kogle, M.E. , Jarczynska, Z.D. , Lehmbeck, J. , Klitgaard, D.K. , and Mortensen, U.H. (2018) Efficient oligo nucleotide mediated CRISPR‐Cas9 gene editing in *Aspergilli* . Fungal Genet Biol 115: 78–89. 29325827 10.1016/j.fgb.2018.01.004

[mbt214033-bib-0034] Oh, B.R. , Seo, J.W. , Heo, S.Y. , Luo, L.H. , Hong, W.K. , Park, D.H. , and Kim, C.H. (2013) Efficient production of 1,3‐propanediol from glycerol upon constitutive expression of the 1,3‐propanediol oxidoreductase gene in engineered *Klebsiella pneumoniae* with elimination of by‐product formation. Bioprocess Biosyst Eng 36: 757–763. 23361186 10.1007/s00449-013-0901-y

[mbt214033-bib-0035] Prykhozhij, S.V. , Caceres, L. , and Berman, J.N. (2017) New developments in CRISPR/Cas‐based functional genomics and their implications for research using zebrafish. Curr Gene Ther 17: 286–300. 29173171 10.2174/1566523217666171121164132

[mbt214033-bib-0036] Przystalowska, H. , Lipinski, D. , and Slomski, R. (2015) Biotechnological conversion of glycerol from biofuels to 1,3‐propanediol using *Escherichia coil* . Acta Biochim Pol 62: 23–34. 25710056 10.18388/abp.2014_885

[mbt214033-bib-0037] Saifaldeen, M. , Al‐Ansari, D.E. , Ramotar, D. , and Aouida, M. (2021) Dead Cas9‐sgRNA complex shelters vulnerable DNA restriction enzyme sites from cleavage for cloning applications. Crispr Journal 4: 275–289. 33876957 10.1089/crispr.2020.0134

[mbt214033-bib-0038] Sankaranarayanan, M. , Seol, E. , Kim, Y. , Chauhan, A. , and Park, S. (2017) Measurement of crude‐cell‐extract glycerol dehydratase activity in recombinant *Escherichia coli* using coupled‐enzyme reactions. J Ind Microbiol Biotechnol 44: 477–488. 28093656 10.1007/s10295-017-1902-7

[mbt214033-bib-0039] Schwartz, C. , Curtis, N. , Lobs, A.‐K. , and Wheeldon, I. (2018) Multiplexed CRISPR activation of cryptic sugar metabolism enables yarrowia lipolytica growth on cellobiose. Biotechnol J 13. 10.1002/biot.201700584 29729131

[mbt214033-bib-0040] Sedlar, K. , Vasylkivska, M. , Musilova, J. , Branska, B. , Provaznik, I. , and Patakova, P. (2021) Phenotypic and genomic analysis of isopropanol and 1,3‐propanediol producer *Clostridium diolis* DSM 15410. Genomics 113: 1109–1119. 33166602 10.1016/j.ygeno.2020.11.007

[mbt214033-bib-0041] Sun, S.Q. , Wang, Y.K. , Shu, L. , Lu, X.Y. , Wang, Q.H. , Zhu, C.G. , *et al*. (2021) Redirection of the central metabolism of *Klebsiella pneumoniae* towards dihydroxyacetone production. Microb Cell Factories 20: 1–16. 10.1186/s12934-021-01608-0 PMC8243499 34187467

[mbt214033-bib-0042] Sun, Y.Q. , Zheng, Y.F. , Wang, X.L. , Zhou, J.J. , and Xiu, Z.L. (2019) Fermentation performance and mechanism of a novel microbial consortium DUT08 for 1,3‐propanediol production from biodiesel‐derived crude glycerol under non‐strictly anaerobic conditions. Process Biochem 83: 27–34.

[mbt214033-bib-0043] Van der Vossen, J. , van der Lelie, D. , and Venema, G. (1987) Isolation and characterization of *Streptococcus cremoris* Wg2‐specific promoters. Appl Environ Microbiol 53: 2452–2457. 2447829 10.1128/aem.53.10.2452-2457.1987 PMC204128

[mbt214033-bib-0044] Velegzhaninov, I.O. , Belykh, E.S. , Rasova, E.E. , Pylina, Y.I. , Shadrin, D.M. , and Klokov, D.Y. (2020) Radioresistance, DNA damage and DNA repair in cells with moderate overexpression of RPA1. Front Genet 11: 1–10. 32849834 10.3389/fgene.2020.00855 PMC7411226

[mbt214033-bib-0045] Wang, P. , Yin, Y. , Wang, X. , and Wen, J.P. (2021a) Enhanced ascomycin production in *Streptomyces hygroscopicus* var. ascomyceticus by employing polyhydroxybutyrate as an intracellular carbon reservoir and optimizing carbon addition. Microb Cell Fact 20: 1–16. 33731113 10.1186/s12934-021-01561-y PMC7968196

[mbt214033-bib-0046] Wang, W.J. , Yu, X. , Wei, Y.J. , Ledesma‐Amaro, R. , and Ji, X.J. (2021) Reprogramming the metabolism of *Klebsiella pneumoniae* for efficient 1,3‐propanediol production. Chem Eng Sci 236: 116539.

[mbt214033-bib-0047] Wang, X. , Zhang, L. , Chen, H. , Wang, P. , Yin, Y. , Jin, J. , *et al*. (1800) Rational proteomic analysis of a new domesticated *Klebsiella pneumoniae* x546 producing 1,3‐Propanediol. Front Microbiol 12: 770109. 10.3389/fmicb.2021.770109 PMC8662357 34899654

[mbt214033-bib-0048] Wei, D. , Wang, M. , Jiang, B. , Shi, J.P. , and Hao, J. (2014) Role of dihydroxyacetone kinases I and II in the dha regulon of *Klebsiella pneumoniae* . J Biotechnol 177: 13–19. 24583287 10.1016/j.jbiotec.2014.02.011

[mbt214033-bib-0049] Wood, B.E. , Yomano, L.P. , York, S.W. , and Ingram, L.O. (2005) Development of industrial‐medium‐required elimination of the 2,3‐butanediol fermentation pathway to maintain ethanol yield in an ethanologenic strain of *Klebsiella oxytoca* . Biotechnol Prog 21: 1366–1372. 16209539 10.1021/bp050100e

[mbt214033-bib-0050] Yun, J.H. , Zabed, H.M. , Zhang, Y.F. , Parvez, A. , Zhang, G.Y. , and Qi, X.H. (2021) Co‐fermentation of glycerol and glucose by a co‐culture system of engineered *Escherichia coli* strains for 1,3‐propanediol production without vitamin B‐12 supplementation. Biores Technol 319: 116539. 10.1016/j.biortech.2020.124218 33049440

[mbt214033-bib-0051] Zhang, Q.R. , and Xiu, Z.L. (2009) Metabolic pathway analysis of glycerol metabolism in *Klebsiella pneumoniae* incorporating oxygen regulatory system. Biotechnol Prog 25: 103–115. 19224565 10.1002/btpr.70

[mbt214033-bib-0052] Zhao, L. , Ma, X.Y. , Zheng, Y. , Zhang, J.G. , Wei, G.D. , and Wei, D.Z. (2009) Over‐expression of glycerol dehydrogenase and 1,3‐propanediol oxidoreductase in *Klebsiella pneumoniae* and their effects on conversion of glycerol into 1,3‐propanediol in resting cell system. J Chem Technol Biotechnol 84: 626–632.

[mbt214033-bib-0053] Zhao, L. , Zheng, Y. , Ma, X.Y. , and Wei, D.Z. (2009) Effects of over‐expression of glycerol dehydrogenase and 1,3‐propanediol oxidoreductase on bioconversion of glycerol into 1,3‐propanediol by *Klebsiella pneumoniae* under micro‐aerobic conditions. Bioprocess Biosyst Eng 32: 313–320. 18682988 10.1007/s00449-008-0250-4

[mbt214033-bib-0054] Zheng, P. , Wereath, K. , Sun, J.B. , van den Heuvel, J. , and Zeng, A.P. (2006) Overexpression of genes of the dha regulon and its effects on cell growth, glycerol fermentation to 1,3‐propanediol and plasmid stability in *Klebsiella pneumoniae* . Process Biochem 41: 2160–2169.

[mbt214033-bib-0055] Zhou, J. , Gao, L. , Liu, L. , Zhang, S. , Fu, S. , and Gong, H. (2014) Influence of blocking formation of D‐lactic acid on 1,3‐propanediol fermentation by *Klebsiella pneumoniae* . Indust Microbiol 44: 24–27.

[mbt214033-bib-0056] Zhu, C.Q. , Jiang, X. , Zhang, Y.Q. , Lin, J. , Fu, S.L. , and Gong, H. (2015) Improvement of 1,3‐propanediol production in *Klebsiella pneumoniae* by moderate expression of puuC (encoding an aldehyde dehydrogenase). Biotech Lett 37: 1783–1790. 10.1007/s10529-015-1851-z 25957564

