# Supplementary material for: Enhancing the capability of Klebsiella pneumoniae to produce 1, 3‐propanediol by overexpression and regulation through CRISPR‐dCas9
Source: Microb Biotechnol. 2022 Mar 17;15(7):2112–25. doi: 10.1111/1751-7915.14033 (PMC9249332; doi:10.1111/1751-7915.14033)
Supplement: Supplementary file 1 — Appendix S1. The microorganism and cultivations. [file MBT2-15-2112-s001.docx]

Enhancing the capability of *Klebsiella pneumoniae* to produce 1,3-propanediol by overexpression and regulation through CRISPR-dCas9

Xin Wang^1,2,4,5^, Lin Zhang^3^, Shaoxiong Liang^1,2^, Ying Yin^1,2^, Pan Wang^1,2^, Yicao Li^1,2^, Wee Shong Chin^5^, Jianwei Xu^4,5^ and Jianping Wen^1,2^*

^1^ Key Laboratory of Systems Bioengineering (Ministry of Education), Tianjin University, Tianjin 300072, P. R. China

^2^ SynBio Research Platform, Collaborative Innovation Center of Chemical Science and Engineering (Tianjin), School of Chemical Engineering and Technology

, Tianjin University, Tianjin 300072, P. R. China

^3^ Dalian Petrochemical Research Institute of Sinopec, Dalian 116000, P. R. China

^4^ Institute of Materials Research and Engineering, #08-03, 2 Fusionopolis Way, Agency for Science, Technology and Research, Singapore 138634

^5^ Department of Chemistry, National University of Singapore, 3 Science Drive 3, Singapore 117543

* Correspondence author: Jianping Wen

Telephone: +86-022-27892061;

Fax: +86-022-27892061;

E-mail: jpwen@tju.edu.cn

**Materials and methods**

**Microorganism and cultivations**

The seed and solid medium (pH 7.0) contained 40 g/L glycerol, 4.12 g/L NH_4_Cl, 0.55 g/L KCl, 0.97 g/L NaH_2_PO_4_·2H_2_O, 0.29 g/L Na_2_SO_4_, 0.26 g/L MgCl_2_·6H_2_O, 0.39 g/L citric acid, 0.93 g/L yeast extract, 0.16 g/L Vc and 4 mL of nutrient solution. Nutrient solution contained 0.037 g/L Na_2_MoO_4_, 0.025 g/L ZnCl_2_, 0.28 g/L CoCl_2_·6H_2_O, 0.151 g/L MgSO_4_·7H_2_O, 0.032 g/L NiCl_2_·6H_2_O and 1.0 mL HCl.

The production medium was a little different from the seed medium, which contained 40 g/L glycerol, 6.20 g/L NH_4_Cl, 0.85 g/L KCl, 1.39 g/L NaH_2_PO_4_·2H_2_O, 0.33 g/L Na_2_SO_4_, 0.31 g/L MgCl_2_·6H_2_O, 1.07 g/L citric acid, 1.14 g/L yeast extract, 0.12 g/L Vc, 0.22 g/L C_5_H_11_NO_2_ and 5 mL of nutrient solution. Nutrient solution contained 5.42 g/L FeCl_3_·6H_2_O, 0.005 g/L Na_2_MoO_4_, 0.05 g/L ZnCl_2_, 0.18 g/L MnCl_2_·4H_2_O, 0.48 g/L CoCl_2_·6H_2_O, 0.065 g/L H_3_BO_4_, 0.679 g/L CuSO_4_·5H_2_O and 1.0 mL HCl.

The seed was continuous operation in 250 mL flask which containing a 100 mL seed medium at 150 rpm for 8.5 h at 37°C. The production of 1,3-PDO was carried out in a 250 mL flask with 100 mL working volume at 150 rpm for 48 h at 37 °C and in a 7.5 L BioFlo 110 fermenter (New Brunswick Scientific, Edison, NJ, USA) at 400 rpm for 40 h at 37°C after adding 770 g glycerol (with a final 5.4 L working volume). Three biological replicates were used for each fermentation experiment. The pH of the seed medium and fermentation medium was adjusted to 7.0 with 3.125 M Na_2_CO_3_ solution respectively.
